# Dual-task motor performance with a tongue-operated assistive technology compared with hand operations

**DOI:** 10.1186/1743-0003-9-1

**Published:** 2012-01-13

**Authors:** Ashley N Johnson, Xueliang Huo, Maysam Ghovanloo, Minoru Shinohara

**Affiliations:** 1 School of Electrical and Computer Engineering, Georgia Institute of Technology, Atlanta, GA, USA; 2 School of Applied Physiology, Georgia Institute of Technology, Atlanta, GA, USA; 3 Rehabilitation R&D Center of Excellence, Atlanta VA Medical Center, Decatur, GA, USA

**Keywords:** dual task, assistive device, motor control, finger

## Abstract

**Background:**

To provide an alternative motor modality for control, navigation, and communication in individuals suffering from impairment or disability in hand functions, a Tongue Drive System (TDS) has been developed that allows for real time tracking of tongue motion in an unobtrusive, wireless, and wearable device that utilizes the magnetic field generated by a miniature disk shaped magnetic tracer attached to the tip of the tongue. The purpose of the study was to compare the influence of a concurrent motor or cognitive task on various aspects of simple movement control between hand and tongue using the TDS technology.

**Methods:**

Thirteen young able-bodied adults performed rapid and slow goal-directed movements of hand and tongue (with TDS) with and without a concurrent motor (hand or tongue) or cognitive (arithmetic and memory) task. Changes in reaction time, completion time, speed, correctness, accuracy, variability of displacement, and variability of time due to the addition of a concurrent task were compared between hand and tongue.

**Results:**

The influence of an additional concurrent task on motor performance was similar between the hand and tongue for slow movement in controlling their displacement. In rapid movement with a concurrent motor task, most aspects of motor performance were degraded in hand, while tongue speed during rapid continuous task was maintained. With a concurrent cognitive task, most aspects of motor performance were degraded in tongue, while hand accuracy during the rapid discrete task and hand speed during the rapid continuous task were maintained.

**Conclusion:**

Rapid goal-directed hand and tongue movements were more consistently susceptible to interference from concurrent motor and cognitive tasks, respectively, compared with the other movement.

## Background

Hand motor functions are essential in daily life, including occupational tasks, because the use of the hands plays critical roles in control, navigation, and communication. In individuals who have impaired or disabled motor functions in one of the hands (*e.g*., unilateral amputation, hemiplegia, and incomplete spinal cord injury), an alternative to the hand is required for performing concurrent motor tasks that are usually accomplished with two hands in able-bodied individuals. For example, they would need a functional actuator that can issue a series of commands (*e.g*. pressing a series of keys, drawing a line) while the able hand is manipulating an object. The speed, accuracy, and variability of performance in the actuator influence the accomplishment of the task. An able foot may be an option for such an alternative, but it is not readily available for an alternative to the hand during standing, walking, or running. Precise control and rapid reactive movement is not suitable for the foot because of the prolonged conduction time due to the distance between the cortex and the foot. In contrast, the tongue appears to have a better potential to serve as a control mechanism and alternative modality for motor function.

**Figure 1:**
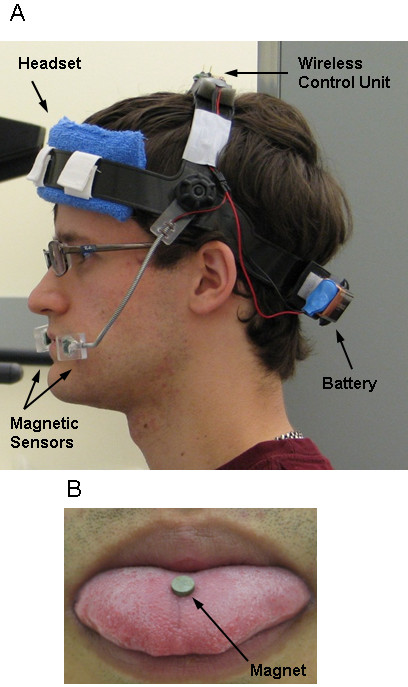
**A subject using the tongue drive system (TDS) to perform tongue tasks**. The major components of the TDS headset are depicted (a). The wireless control unit sits on top of the headset with the battery supply in the back. Two magnetic sensors are attached (one on each side) to detect the movement of the magnet on the subject's tongue (b).

The tongue occupies a considerable area of the sensory motor cortex, comparable with that of the hand [1]. The tongue is connected to the brain via the hypoglossal nerve over a shorter distance compared with hand and finger that are connected through the spinal cord. The tongue's motion in the mouth is rapid and intuitive. Training the tongue with a simple protrusion task is reported to induce neural plasticity [2]. Accordingly, the tongue appears to be appropriate as a new control interface. To this end, a few tongue-operated assistive technologies, such as the Tongue-Touch-Keypad [3], Jouse2 [4] and Integra Mouse [5], have been developed. However, these technologies are limited by their large size, requirements for specific head movement, and potential for causing fatigue. In contrast, a recently developed Tongue Drive System (TDS) (Figure 1) allows for real time tracking of tongue motion without these limitations because it detects tongue motion with magnetic field around the mouth, which is generated by a small magnetic tracer attached to the tongue, with an array of magnetic sensors mounted on a light-weight headset. Hence, the TDS is an unobtrusive, noninvasive, wireless, tongue-operated assistive technology that can potentially substitute some of the hand functions with tongue motions [6]. The architecture and performance evaluation of the TDS by able-bodied and disabled subjects have been reported elsewhere [6-10].

Currently, the cost and benefits of using the tongue as a new modality for motor functions in human-system integration is unclear. So far, most studies on the tongue's motor abilities are on natural tongue functions such as respiration, swallowing, and speech [11,12]. Recently, voluntary tongue protrusion and a static holding maneuver were employed to characterize neuromuscular activity of tongue muscles [13-15]. However, characteristics of tongue-motor performance during goal-directed voluntary motor control are unknown. Direct comparison of motor performance between tongue and other actuators (*e.g*. hand) is technically challenging because of difficulties in designing exact same tasks and measurements between them. In addition, the measurements on tongue motor performance may be influenced by the inherent characteristics of the employed tongue-operated technology.

In the real life, a tongue-operated technology such as TDS would most likely be used with concurrent activities. Motor performance is often degraded with concurrent activities depending on the type of motor task and concurrent motor or cognitive activity [16-18]. Depending upon the concurrent activity, priority may be placed on one of the tasks resulting in a degradation of performance for the task not selected. For example, driving and needing to take a note from talking on the cell phone or jogging and needing to answer the phone each requires a shift in the attentional resources devoted to the concurrent tasks. It is thus important to understand how goal-directed tongue motor performance is influenced by an addition of concurrent activity.

To shed light on voluntary tongue motor control during goal-directed motor tasks, the current study used the TDS to track tongue motion and focused on clarifying the changes in tongue motor performance when a concurrent task is added. It is unknown how hand motor performance is influenced by the addition of concurrent tongue motor task and *vice versa*. Hence, the purpose of the study was to compare the changes in motor performance in hand and tongue with an additional concurrent motor or cognitive task during rapid and slow goal-directed movement in young able-bodied adults. The findings would help us understand the similarities and differences between hand and tongue in the influence of an additional concurrent task on motor performance.

## Methods

### Experimental Design

Subjects performed experimental trials in 3 sessions. In each session, subjects controlled the movement of a computer cursor that corresponded to the movement of their right index finger or tongue. The sessions were conducted in a random sequence on 3 days that were separated > 7 days for each subject. The current purpose of the study was tested in Sessions 1 and 2 in which motor performance in simple goal-directed tasks was examined that included rapid discrete movements (Session 1) and rapid continuous movements or slow movements (Session 2) of the index finger and the tongue. Although Session 3 was also performed by the same group of subjects, it was conducted for distinct research purposes and experimental setup to clarify the influence of background physical exertion on the hand and tongue motor performance and thus will not be included.

### Subjects

Thirteen healthy young right-hand dominant adults (age: 20-26 years old; 7 men and 6 women) participated in the study. None of them had any history of neurological disorder. Subjects refrained from caffeine and nicotine ~ 3 hours prior to the experiment. Handedness of the subjects was confirmed with the Edinburgh Handedness Inventory [19]. Subject's written consent was obtained in accordance with the Institutional Review Board of Georgia Institute of Technology and the U.S. Army Research Office.

### TDS setup

In all experiments, subjects wore a TDS headset (Figure 1A), and TDS setup was performed before experiments. The magnetic sensors of the TDS, which were mounted on a pair of goosenecks, were positioned close to the subjects' cheeks so the sensors detect variations of the magnetic field resulting from different positions of the magnetic tracer in the mouth with a high signal to noise ratio. Subjects walked around the test setup to let the sensor signal processing (SSP) algorithm identify the background magnetic noise (primarily from the earth's magnetic field) which needed to be cancelled out during the experiments. Then a small (∅5 mm, 1.5 mm thick) nickel plated rare-earth permanent magnet (K&J Magnetics, Jamison, PA) with 13500 Gauss residual flux density was attached near the tip of the subjects' tongue using Cyanodent dental adhesive (Ellman International, Oceanside, NY) (Figure 1B). The position of the magnet on the subjects' tongue was noted by marking to ensure that they could be reattached in the same positions if detached from the subjects' tongue. There is no risk of swallowing the magnet because subjects can easily detect when the magnet is getting loose.

The TDS prototype can detect up to six user-defined "tongue commands", which have been originally assigned to cardinal cursor movements and single/double clicks to substitute computer mouse functions [10]. Before using these commands, subjects were required to define them for the SSP algorithm in a calibration session [9]. This was accomplished by identifying specific positions for the tip of the tongue in their mouth and associating them to each "tongue command". For instance, subjects may touch the lower left canine teeth with the tip of their tongue and define it as the LEFT command. During the calibration session, subjects were instructed to consistently place their tongue at 4 recommended positions that were assigned to LEFT, RIGHT, UP, and DOWN commands in a sequence, 10 times in a row, such that TDS would collect enough magnetic sensor data for those specific commands and extract the principal features for those commands based on the recorded data. Later on, when subjects placed their tongue at those specific positions, TDS correctly associated those positions to the tongue commands.

### Session 1: Rapid discrete movement

Reaction time, completion time, and correctness were measured in multiple tasks that required rapid discrete movements. Session 1 was comprised of 6 tasks: simple hand (H), simple tongue (T), simple cognitive (C), concurrent hand and tongue (HT), concurrent hand and cognitive (HC), and concurrent tongue and cognitive (TC) tasks. Each task had 3 difficulty levels with 1, 2, or 4 choices in a randomized order. Subjects repeated the 30-s trial 3 times in each level of each task after a practice trial. Subjects wore the TDS headset and sat in a chair with their right arm resting on an adjacent table, 1.5 m away from a 22-inch monitor with 1600 × 1200 resolution. The monitor showed a customized graphical user interface (GUI) providing visual cues and feedback on the task being performed. The following hand and tongue tasks were designed in a way that their task requirements resembled each other as much as possible. Subjects performed H, T, C, HT, HC, and TC tasks in a randomized order. There was no specific instruction to prioritize either task during the concurrent tasks.

#### Hand task

**Figure 2:**
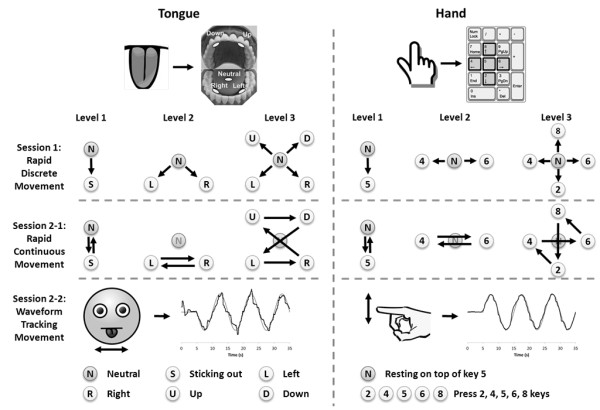
**Cartoon schematic to explain the experimental conditions for Session 1 (Rapid Discrete Movement), Session 2 part 1 (Rapid Continuous Movement), and Session 2 part 2 (Waveform Tracking Movement)**. A representative cursor trajectory for the waveform tracking a sinusoid is included for Session 2 part 2.

The hand task was to press a key on a numeric keypad with the right index finger following a visual cue (Figure 2). Subjects lightly rested their right index finger on top of the home key ('5') in the center of the numeric keypad without pressing it. On the monitor, three lights turned on from red, to yellow, and to green in 1 s intervals at the beginning of each trial indicating for subjects to be ready. When the green light turned off, a pink command indicator turned on as the visual cue next to the designated command to initiate the task as soon as possible.

In the Level 1 hand task, subjects were instructed to press the home key ('5') for 0.5 second as soon and as quickly as they recognized the pink indicator (simple reaction task). As visual feedback, the length of the horizontal bar next to the indicator increased while the correct key ('5') was being pressed (Figure 2). The length of the bar indicated the duration of the key pressing task. When the bar was filled and the completion indicator was lit up, subjects released the key and rested their finger on top of the home key ('5'). The Level 2 hand task was a choice reaction task with 2 choices. The same procedure was followed as in Level 1 except that subjects were instructed to press key '8' (above the home key) in response to the UP command indicator and key '2' (below the home key) in response to DOWN command indicator (Figure 2). In each trial, one of the 2 indicators turned on randomly, as a visual cue, and subjects were instructed to choose and press the associated key as quickly and accurately as possible. The Level 3 hand task was a choice reaction task with 4 choices. The subjects were instructed to press key '8' (above the home key), key '6' (right side of the home key), key '2' (below the home key), and key '4' (left side of the home key) in response to the UP, RIGHT, DOWN, and LEFT command indicators, respectively (Figure 2).

In all hand tasks, if the correct key was not pressed within 1.5 s after the command indicator was turned on, the action was regarded as failed. In this case, subjects were instructed to move on to the next command without trying to correct themselves. The command indicator was repeatedly turned on and off with pseudo random time intervals (randomized between 0.5-1.0 s) for 20 times. The correctness of completed hand task was expressed as the percentage of correctly completed actions out of all executed actions. The timing of the pressed key was detected and saved for later processing. To detect the onset of finger movement, a miniature single-axis accelerometer (A352C65, mass: 2 g, diameter: 9.5 mm, PCB Piezotronics, Depew, NY) was attached to the index finger in between the dorsal and proximal interphalangeal joints with a double-sided adhesive tape. The acceleration signal was amplified, digitized, and recorded using Power 1401 and Spike2 (Cambridge Electronic Design, Cambridge, UK) at a sampling frequency of 1 kHz.

#### Tongue task

Tongue tasks and their associated GUIs were deliberately designed similar to the H tasks in a way that the feedback of individual hand and tongue performances was comparable. Holding the tongue in its natural resting position was equivalent to resting the index finger on the home key ('5'). In the Level 1 tongue task, sticking the tongue out corresponded to pressing key '5' in the Level 1 hand task (Figure 2). In the Level 2 tongue task, touching the lower left and lower right teeth with the tip of the tongue corresponded to pressing keys '8' and '2', respectively, in the Level 2 hand task (2 choices) (Figure 2). In the Level 3 tongue task, touching the lower left, upper left, lower right, and upper right teeth with the tip of the tongue corresponded to pressing keys '4', '8', '6', and '2', respectively, in the Level 3 hand task (4 choices) (Figure 2).

#### Cognitive task

Cognitive task required subjects to perform a cumulative math calculation. Subjects were given 10 math operations verbally during the 30 s trial period. The math operations included single digit summation, subtraction, and multiplication not exceeding 100. The math problems were randomly generated and recorded in computer audio files, which were replayed in randomized sequence during the experiment. Subjects were asked only one math problem at a time and memorized the result of the previous calculation that was carried over to the next operation. Subjects reported only the final and accumulated answer immediately after the 30-s trial ended.

#### Data analysis

Reaction time of the motor (hand and tongue) tasks, completion time of the motor tasks, and correctness of motor and cognitive tasks were determined. Reaction time was measured as the duration between the appearance of the visual cue and the onset of movement in the finger or tongue. The onset of movement was determined visually from the abrupt large change in the signal from the accelerometer on the finger or from the magnetic sensors on the TDS. Completion time was measured as the duration between the appearance of the visual cue and the correct completion of the task. Completion times were not calculated for the tasks that were not correctly performed (*e.g*. when subjects pressed wrong keys). Correctness of the motor task was measured as the number of correctly completed trials out of all the executed trials, and expressed in percentage. Correctness of the cognitive task was measured as the number of correctly answered problems out of all the answered problems, and expressed in percentage.

#### Statistical analysis

Obtained variables were averaged across 3 trials in each task. The dependent variables were reaction time, coefficient of variation (CV) of reaction time, completion time, and correctness. Statistical significance about the effects of an additional task and difficulty level (Levels 1-3) on these dependent variables was tested with a two-way analysis of variance (ANOVA) with repeated measures. Post-hoc analyses were performed with a Bonferonni test when appropriate. An alpha level of 0.05 was used for all statistical comparisons, and *P *< 0.05 or *P *< 0.01 was indicated when significance was found. Unless stated otherwise, the data are presented as mean ± standard deviation (SD) in the text and table and mean ± standard error (SE) in figures.

### Session 2: Rapid continuous movement and slow waveform tracking movement

Session 2 compared speed, correctness, accuracy, variability of time, and variability of displacement in multiple tasks that required continuous and continuous movements of the tongue or finger. This session was broken into two parts: rapid continuous movements (Part 1) and slow waveform tracking movements (Part 2). In each session, subjects performed H, T, C, HT, HC, TC tasks in a randomized order. There was no specific instruction to prioritize either task during the concurrent tasks.

### Session 2, Part 1: Rapid continuous movement

Subjects performed tasks similar to Session 1 except for being instructed to repeat the movement continuously for 15 s as fast and quickly as possible. Three 15-s trials were performed for each level of the task, and subjects practiced each finger/tongue task up to 2 times before the actual trials. The order of the tasks was randomized.

#### Hand task

In Level 1 hand task, subjects were instructed to press and release the home key ('5') continuously as fast and accurately as possible for the duration of the task. In Level 2 hand task, the same procedure was followed except that subjects were instructed to begin at a resting position (index finger over home key '5') and then move their index finger and press and release, in sequence, key '8' (above key '5'), then key '2' (below key '5') repeatedly, as fast and accurately as possible. In Level 3 hand task, subjects were instructed to begin at the resting position and press and release, in sequence, number keys '8' (above key '5'), '2' (below key '5'), '4' (left of key '5'), and '6' (right of key '5') repeatedly with their index finger, as fast and accurately as possible.

#### Tongue task

Tongue task was performed in a similar way to Session 1, except that subjects were asked to move the tongue between the target positions in their mouth as fast and accurately as possible for 15 s. In each difficulty level, the tongue moved to a position similar to the finger pressing a key in the hand tasks. All sequences for Levels 1, 2 and 3 remained constant, as well. Visual feedback was provided via a green light to indicate whether subjects had moved the tongue to the correct position. When an incorrect tongue command was issued, subjects were instructed not to try to correct their mistake. Instead, they were asked to continue the sequence. Visual feedback was provided in a similar manner for the hand task.

#### Cognitive task

The cognitive task was consistent with Session 1 except that subjects were given 5 math operations during the 15-s trial period.

### Session 2, Part 2: Slow waveform tracking movement

Subjects tracked a step waveform and a sinusoidal waveform. The step waveform consisted of a 30-s step following a 5 s period at the resting level. The sine waveform consisted of 3 cycles of sinusoidal waveforms with a period of 10 s after a 5 s period at the resting level. Three trials were performed for each task and waveform. Subjects practiced each hand/tongue task up to 2 times before the actual trials. The order of the tasks and waveforms was randomized.

#### Hand task

The hand task was to track a waveform steadily and accurately with the finger movement. Subjects' right arm and hand were fixed on a Versa-Form pillow (Tumble Form, Bolingbrook, IL) for support. A small light emitting diode (LED) tracer was attached to the tip of the right index finger to track the movement of the finger in 3-D space using an Optotrak motion capture system (Northern Digital Inc., Ontario, Canada). Sensor outputs from the finger movements were displayed on the GUI in real time to provide visual feedback. Voluntary maximum extension of the index finger was determined by having subjects extend their index finger to maximum extension and recording that value relative to their neutral (level) position. For the step waveform, the amplitude of the waveform was set to 50% of voluntary maximum extension. For the sinusoidal wave, the peak amplitudes of the target sine wave were set to 50% of the voluntary maximum extension. Subjects started with their index finger level and extended and flexed their finger to track the assigned waveform as steadily and accurately as possible.

#### Tongue task

Combined sensor outputs from TDS were recorded after noise cancellation and used to represent the position of the tongue. To determine the maximum and minimum sensor output, the magnetic field was recorded while subjects moved their tongue to an extreme position on the left and right, *i.e*. touching the edge of their lips, near the left and right cheeks. For the square wave, the amplitude of the target waveform was set to 50% of the maximum sensor output. Subjects were asked to adjust the position of their tongue with visual feedback, *i.e*., moving them closer to or further away from the left sensor module. For the sinusoidal wave, the peak amplitudes of the target waveform were set to 50% of the maximum and minimum sensor outputs when the tongue was at the two extreme positions.

#### Cognitive task

This task was similar to the cognitive task for Part 1 with two differences. First, 10 cognitive iterations were performed for each trial with the final answer to the iterations requested at the conclusion of the trial. Second, cognitive problems were not given until after the initial 5 s of the level holding period of the waveforms. Thus the cognitive task was only performed during the actual tracking of the step or sinusoidal waveforms.

#### Data analysis

Speed, correctness, accuracy, variability of displacement and variability of time were determined. In Part 1, speed was assessed by the frequency of motion defined as the total counts divided by the trial duration (15 s). Motor correctness for Part 1 was determined by dividing the correct number of patterns (*i.e*. sequences) executed by the total number of patterns. Variability of time interval was measured as the CV of time interval. The time interval was defined as the time between key presses or tongue movements. In Part 2, the performance metric of accuracy was evaluated by the absolute error of the waveform tracking excluding the resting portion. Variability of displacement was measured as the root mean square error (RMSE) for the waveform tracking. The calculated variables were averaged across 3 trials in each task.

#### Statistical analysis

Dependent variables were speed (frequency of motion), correctness, absolute error, RMSE, and the CV of time interval. These variables were tested with a univariate approach for two-way ANOVA with repeated measures. A Greenhouse-Geisser correction was used for the cases where the Sphericity assumption was not met based on Mauchly's test. The factors were task and difficulty level (Levels 1-3). A one-way ANOVA was applied to determine the significance for cognitive correctness. Post-hoc analyses were performed with a Bonferonni test when appropriate. An alpha level of 0.05 was used for all statistical comparisons, and *P *< 0.05 or *P *< 0.01 was indicated when significance was found. Unless stated otherwise, the data are presented as mean ± SD in the text and table and mean ± SE in figures. A summary of statistical results with ANOVAa on the main effect and interaction are presented in Table 1.

**Table 1:** Summary of statistical results with ANOVAs on the main effect and interaction

Session 1				
	**Hand**		**Tongue**	
	
	***p***	**Observed power**	***p***	**Observed power**

**Reaction time**				

*Difficulty level*	< 0.001 *	0.994	< 0.001 *	1.000
*Task*	< 0.001 *	1.000	0.003 *	0.910
*Difficulty level × Task*	< 0.001 *	0.999	0.031 *	0.682

***CV of reaction time***				

*Difficulty level*	0.002 *	0.935	0.067	0.539
*Task*	0.018 *	0.740	< 0.001 *	0.999
*Difficulty level × Task*	0.070	0.631	0.128	0.439

***Completion time***				

*Difficulty level*	< 0.001 *	1.000	< 0.001 *	1.000
*Task*	< 0.001 *	1.000	< 0.001 *	1.000
*Difficulty level × Task*	< 0.001 *	1.000	< 0.001 *	1.000

***Motor correctness***				

*Difficulty level*	0.009 *	0.809	< 0.001 *	1.000
*Task*	< 0.001 *	0.998	< 0.001 *	1.000
*Difficulty level × Task*	0.003 *	0.923	< 0.001 *	1.000

***Session 2, Part 1***				

	Hand		Tongue	
	
	*p*	Observed power	*p*	Observed power

***Speed***				

*Difficulty level*	< 0.001 *	1.000	0.066	0.536
*Task*	< 0.001 *	1.000	0.019 *	0.733
*Difficulty level × Task*	< 0.001 *	0.998	0.003 *	0.922

***CV of time interval***				

*Difficulty level*	< 0.001 *	1.000	< 0.001 *	1.000
*Task*	0.491	0.160	0.004 *	0.901
*Difficulty level × Task*	< 0.001 *	1.000	0.072	0.626

***Motor correctness***				

*Difficulty level*	< 0.001 *	1.000	< 0.001 *	1.000
*Task*	0.012 *	0.771	< 0.001 *	0.999
*Difficulty level × Task*	< 0.009 *	0.812	< 0.001 *	1.000

***Session 2, Part 2***				

***RSME***				

*Waveform*	< 0.001 *	1.000	0.001 *	0.966
*Task*	0.255	0.200	0.017 *	0.748
*Waveform × Task*	0.001 *	0.978	0.434	0.128

*, *P *< 0.05

## Results

Presentation of results is mainly focused on the differences between hand and tongue in the main effects.

### Session 1: Rapid discrete movement

**Figure 3:**
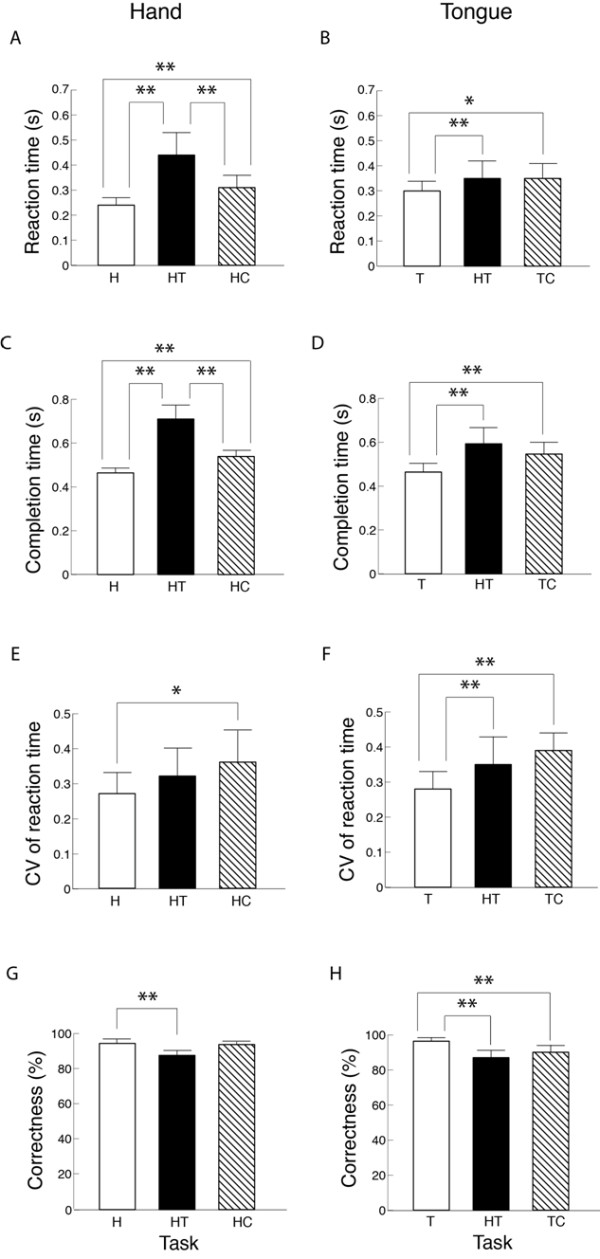
**Main effects of task on reaction time, completion time, coefficient of variation (CV) of reaction time, and correctness during rapid discrete movement**. The data in hand (a, c) and tongue (b, d) in Session 1 are presented. H, independent hand task; T, independent tongue task; HT, concurrent hand-tongue task; HC, concurrent hand-cognitive task; TC, concurrent tongue-cognitive task. *, *P *< 0.05; **, *P *< 0.01.

The reaction time during the independent task at Level 1 was 204.6 ± 27.4 ms in the hand and 236.0 ± 54.8 ms in the tongue. The completion time during the independent task at Level 1 was 339.3 ± 48.7 ms in the hand and 372.6 ± 71.8 ms in the tongue. The influence of an additional concurrent task was different between the hand and tongue, but similar between the reaction time and completion time. In the hand, the reaction time increased significantly with the concurrent cognitive task by 69 ms and with the concurrent tongue task by 194 ms, resulting in a significantly greater value in the latter (Figure 3A). While the tongue reaction time also increased significantly with the concurrent cognitive task by 57 ms and with the concurrent hand task by 55 ms, the values were not significantly different between the concurrent tasks (Figure 3B). Similar, but greater effects of concurrent task were observed for the completion task (Figures 3C & 3D). Both the reaction time and completion time were significantly longer for choice reaction tasks (Levels 2 & 3) compared with a simple reaction task both in the hand and tongue (Table 2).

**Table 2:** Main effect of difficulty level on reaction time, completion time, variability of reaction time, and correctness during rapid discrete movement

	Hand	Tongue
***Reaction time (s)***		

*Level 1*	0.254 ± 0.076	0.261 ± 0.079
*Level 2*	0.339 ± 0.131**	0.321 ± 0.092**
*Level 3*	0.405 ± 0.202**,^††^	0.422 ± 0.133**

***Completion time (s)***		

*Level 1*	0.377 ± 0.065	0.409 ± 0.097
*Level 2*	0.628 ± 0.133**	0.491 ± 0.117**
*Level 3*	0.708 ± 0.212**	0.704 ± 0.144**

***CV of reaction time***		

*Level 1*	0.378 ± 0.161	0.358 ± 0.109
*Level 2*	0.294 ± 0.119*	0.308 ± 0.098
*Level 3*	0.274 ± 0.125**	0.352 ± 0.127

***Correctness (%)***		

*Level 1*	96.7 ± 4.8	94.7 ± 5.6
*Level 2*	97.6 ± 3.8	97.0 ± 3.4
*Level 3*	91.8 ± 14.5*	81.7 ± 13.8**

Mean ± SD. *, *P *< 0.05 *vs. Level 1*, **, *P *< 0.01 *vs. Level 1 *and ††, *P *< 0.01 *vs. Level 2*.

The CV of reaction time increased with an additional task in both the hand and the tongue, on average, with the significant increasing when the concurrent cognitive task was performed (Figures 3E & 3F). The CV of reaction time decreased significantly in choice reaction tasks (Levels 2 and 3) compared with a simple reaction task (Level 1) in the hand, but not in the tongue (Table 2).

The effect of an additional concurrent task on motor correctness was different between the hand and tongue. In the hand, the correctness decreased significantly only with the concurrent tongue task by 9%, but not with the concurrent cognitive task (Figure 3G). For the tongue, the motor correctness decreased significantly with both the concurrent hand and cognitive tasks similarly (by 7% - 10%) (Figure 3H). Motor correctness decreased significantly only at Level 3 in both the hand by 5% and the tongue by 16% (Table 2).

Cognitive correctness was significantly less with the concurrent tongue task compared with the concurrent hand task (Table 3). There was no statistically significant difference in cognitive correctness between difficulty levels in concurrent hand or tongue task.

**Table 3:** Main effect of task and difficulty level on cognitive correctness

Session 1						
	**Task**		**Difficulty level**			
	
	***HC***	***TC***	***1***	***2***		***3***
	
	54.8 ± 32.1	41.9 ± 33.2*	56.5 ± 28.1	51.3 ± 36.9		37.2 ± 31.9
*Control*	53.9 ± 29.2					
**Session 2**						

	**Task**		**Difficulty level**			
	
	***HC***	***TC***	***1***	***2***		***3***

*Part 1*	78.6 ± 29.1	70.1 ± 38.8	74.4 ± 34.4	82.1 ± 30.2		66.7 ± 37.7
*Part 2*	65.4 ± 25.8†	48.7 ± 28.6^†^,*	***Square***		***Sinusoidal***	
			62.8 ± 23.7		51.3 ± 31.6	
*Control*	87.2 ± 16.9					

Mean ± SD (*n *= 13) in%. H, independent hand task; T, independent tongue task; HT, concurrent hand-tongue task; HC, concurrent hand-cognitive task; TC, concurrent tongue-cognitive task. *, *P *< 0.05 *vs*. HC; †, *P *< 0.01 *vs*. Control

### Session 2, Part 1: Rapid continuous movement

**Figure 4:**
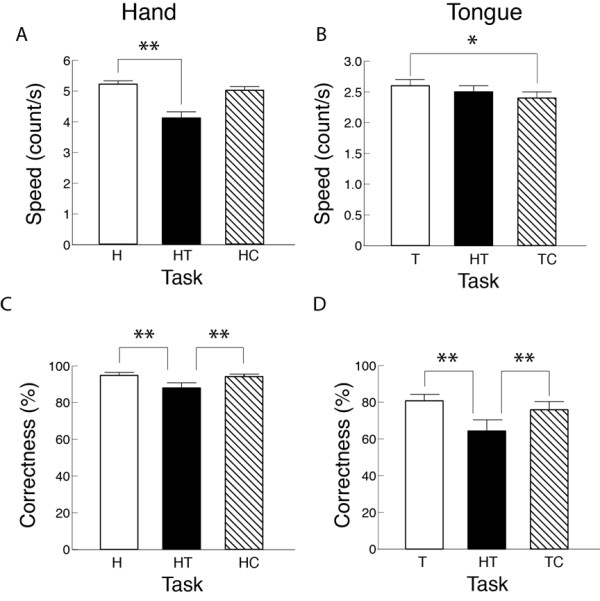
**Main effects of task on the speed and correctness of movement during rapid continuous movement**. The frequency of movement in hand (a, c) and tongue (b, d) in Part 1 of Session 2 is presented in counts/s. H, independent hand task; T, independent tongue task; HT, concurrent hand- tongue task; HC, concurrent hand-cognitive task; TC, concurrent tongue-cognitive task. *, *P *< 0.05; **, *P *< 0.01.

The speed of rapid continuous movement during the independent task at Level 1 was 6.0 ± 0.9 counts/s in hand and 2.3 ± 0.7 counts/s in tongue. The influences of task and difficulty level on the speed were different between the hand and the tongue. In the hand, speed did not change significantly with the concurrent cognitive task, but decreased significantly by 26% with the concurrent tongue task (Figure 4A) compared with the independent task. While the hand speed decreased significantly with difficulty level, the decline with the concurrent tongue task was less obvious at Level 2 (data not shown) in which tongue speed was highest (Table 4). In contrast to hand speed, the continuous tongue speed did not change significantly with the concurrent hand task, but decreased significantly with the concurrent cognitive task by 5% (Figure 4B).

**Table 4:** Main effect of difficulty level on speed and correctness during rapid continuous movement

	Hand	Tongue
***Speed (count/s)***		

*Level 1*	5.53 ± 0.93	2.29 ± 0.65
*Level 2*	4.66 ± 0.48**	2.82 ± 0.80
*Level 3*	4.17 ± 1.17^**,††^	2.50 ± 0.51

***Correctness (%)***		

*Level 1*	100.0 ± 0.0	97.2 ± 8.4
*Level 2*	95.7 ± 3.4**	80.5 ± 22.4**
*Level 3*	80.2 ± 15.1^**,††^	43.3 ± 26.0^**,††^

Mean ± SD. *, *P *< 0.05 *vs. Level 1*, **, *P *< 0.01 *vs. Level 1 *and ††, *P *< 0.01 *vs. Level 2*.

Motor correctness during rapid continuous movements was influenced by the task and difficulty level in a similar manner between the hand and tongue. In both the hand and tongue, motor correctness declined significantly during the concurrent hand-tongue task compared with the independent task (by 7% *vs*. H, by 25% *vs*. T) and the concurrent cognitive task (*vs*. HC or TC) across difficulty levels (Figures 4C & 4D). Motor correctness decreased significantly with increases in the difficulty level in both hand and tongue with apparently greater declines in tongue, on average (Table 4). Cognitive correctness was not significantly influenced by task or difficulty level with the rapid continuous movement (Table 3).

**Figure 5:**
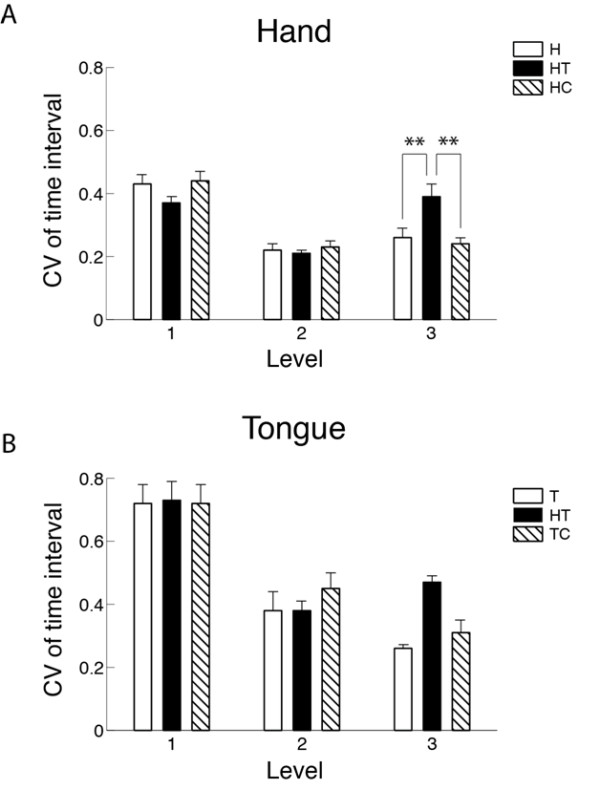
**Effects of task and difficulty level on the coefficient of variation (CV) of time interval during rapid continuous movement**. The data in hand (a) and tongue (b) in Part 1 of Session 2 are presented. H, independent hand task; T, independent tongue task; HT, concurrent hand-tongue task; HC, concurrent hand-cognitive task; TC, concurrent tongue-cognitive task. **, *P *< 0.01.

The significant effect of task on the CV of time interval between rapid continuous key pressings was present only at the most difficult level in the hand. At Level 3, the CV of time interval during the concurrent motor task was significantly greater compared with the independent hand task and concurrent hand and cognitive task (Figure 5A).

### Session 2, Part 2: Slow waveform tracking movement

**Figure 6:**
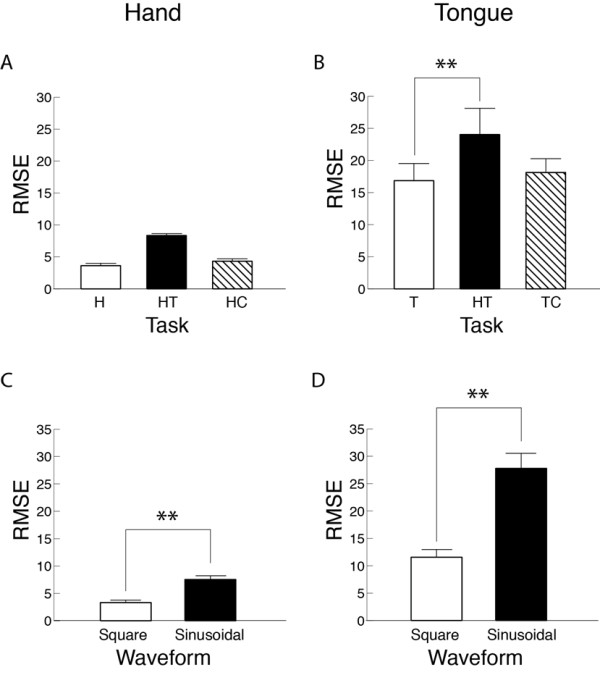
**Main effects of task and difficulty level on the variability of displacement during slow waveform tracking**. The root mean square error (RMSE) in hand (a, c) and tongue (b, d) in Part 2 of Session 2 are presented. Difficulty levels include square-wave and sine wave. H, independent hand task; T, independent tongue task; HT, concurrent hand-tongue task; HC, concurrent hand-cognitive task; TC, concurrent tongue-cognitive task. **, *P *< 0.01.

Variability of displacement during the waveform tracking, as measured by RMSE, was significantly greater for the sinusoidal waveform compared with the square waveform in both the hand (Figure 6C) and tongue (Figure 6D). On average, a concurrent task increased the variability with the concurrent hand-tongue task in both the hand (by 57%, Figure 6A) and tongue (by 30%, Figure 6B), but the significant difference was observed only in the tongue. The relative increase in the average value from the independent to concurrent motor task for the sinusoidal waveform was much greater in the hand (by 60%) compared with the tongue (by 32%) (data not shown). The influences of waveform and task were essentially the same for the accuracy of displacement as measured by the absolute error, with a much greater relative increase in the hand (by 60%) compared with the tongue (by 35%) for the sinusoidal waveform (data not shown).

Cognitive correctness decreased significantly with an additional task for both hand and tongue (Table 3). In particular, cognitive correctness during the concurrent tongue and cognitive task was significantly less compared with the concurrent hand and cognitive task for both waveforms.

## Discussion

The present study revealed that the susceptibility to interferences with an additional concurrent task was different between hand and tongue depending on the additional task. In short, distinct effects of an additional concurrent task between the hand and tongue were observed mostly on motor performance that required a rapid reaction or movement, whereas little distinction was apparent between the effects on the hand and tongue in motor performance that was performed slowly or steadily. Discussion will focus on these major findings.

During rapid discrete movement (Session 1), the concurrent motor task (HT) negatively influenced the speed-related motor performance, *i.e*., reaction time (Figure 3) and completion time (Figure 3), in both the hand and tongue. These negative influences were apparently greater in the hand than in the tongue resulting in significantly greater values during the concurrent motor task compared with the concurrent cognitive task only in the hand. The absence of a significant change in the CV of hand reaction time (Figure 3) may be due to this large increase in the mean reaction time (note CV = standard deviation divided by mean). The amount of increase in reaction time in the hand with a concurrent tongue task ( ~ ×2) was much greater than that with a concurrent motor task by a contralateral hand or foot ( ~ 10%) in the literature [20-23]. The greater influence of concurrent hand-tongue task on the speed-related motor performance in the hand than in the tongue was also supported by the significantly reduced speed only in the hand during rapid continuous movement with the concurrent motor task (Part 1 in Session 2) (Figure 3). It is likely that greater attentional resources were distributed to the tongue than hand task. The results suggested that, for rapid movement, motor performance is less susceptible in tongue than hand to the interference from the concurrent activity of the other motor modality.

The effect of the concurrent cognitive task was in contrast to that of concurrent motor task. In rapid discrete movements (Session 1), the reaction time and completion time increased with a concurrent cognitive task in both hand and tongue. However, the significant reduction in motor correctness was found only in tongue (Figure 3), and the cognitive correctness was reduced only in TC (Table 3). These results indicated that the accuracy of reaction movement tasks and cognitive tasks were more susceptible in tongue than hand to the interference due to the addition of a concurrent cognitive task. This indication in rapid movement appears to hold true whether the movement is discrete or continuous. In rapid continuous movements (Part 1 in Session 2), the concurrent cognitive task significantly reduced speed only in the tongue (Figure 4) without an influence on motor correctness (Figure 4). The absence of a significant difference in cognitive correctness between tongue-cognitive and hand-cognitive tasks (Table 2) implied the greater susceptibility in the tongue to interference from the concurrent cognitive task despite the assumed similar amount of available attentional resources in both the tongue and hand. These results suggested that rapid motor performance is more susceptible in the tongue than in the hand to the interference from a concurrent cognitive task.

The current findings highlight the influence of hand and tongue interaction for performing concurrent tasks. Experimental conditions were set for the confounds of a laboratory setting to provide a baseline for this line of research. Potential extrapolations can be made to individuals who might need assistive technology to perform tasks. The findings of decreased task performance during concurrent tasks for motor-motor and motor-cognitive tasks of the hand and walking [16-18,24] are further extended to the tongue and hand in the present study.

Whereas no report can be found in the literature on goal-directed motor performance of the tongue with respect to the interference from a concurrent task, comparisons to available studies on the neighboring lip motor performance during speech may provide some insights. The greater influence from a concurrent cognitive than a hand task to the speed of tongue movement was contrary to the previous findings on lip movement [25,26]. In both previous studies, velocity of lip movement during speech was reduced with a concurrent hand but not cognitive task. In these studies, the hand task was to manipulate nuts, bolts, and washers without time constraints [26] or to move a mouse and click on a moving object on the computer monitor as often as possible [25]. The cognitive task was to count backwards from 100 by sevens [26] or to solve two-digit math subtraction problems [25]. The current hand and cognitive tasks are practically similar to those in [25] rather than [26]. Although the potential influence of differences in task details cannot be ruled out, the current findings implied that the susceptibility to interference from concurrent hand and cognitive tasks during rapid movement may be different between the tongue and lip despite of their neighboring existence.

For slow or steady movement, the current study found that an additional task influences the hand and tongue similarly with regard to the presence of interference. This is in contrast to the rapid movement described above. When subjects performed a slow steady movement (Session 2, Part 2), a significant effect of an additional concurrent task was found in the same comparison pairs across hand and tongue. In both the hand and tongue, variability and accuracy of displacement increased with an additional motor task using the other motor modality (HT in Figure 6). With an additional cognitive task, the unvarying variability and accuracy of displacement in the slow or steady movement was in contrast to degraded motor performance in rapid movements. In slow finger force control, no or little interference from a concurrent cognitive task has been demonstrated [17,27]. The current results extended this finding in the hand to the tongue and indicated that strategies for performing the slow waveform tracking were less susceptible to interference from an additional cognitive task compared with those for rapid movements in both the hand and tongue. Degradation of task performance with an additional concurrent task occurs most likely because the attentional resources allocated to one or both tasks is reduced when the attentional resources needed to perform two tasks concurrently exceeds the total capacity [28]. Indeed, the absence of a significant increase in variability and accuracy with the concurrent cognitive task (HC or TC) accompanied a reduced cognitive correctness in the tongue-cognitive task compared with the hand-cognitive task. These findings implied that a greater amount of attentional resources were naturally allocated to the tongue task than the hand task.

In general, motor performance was degraded with increased level of task difficulty in both hand and tongue with a couple of exceptions. Tongue speed was faster in Level 2 (*i.e*., continuous horizontal displacement) than Level 1 (*i.e*., continuous protrusion and retraction) in rapid continuous (Table 4; Session 2, Part 1) but not discrete (Table 2; Session 1) movement. Considering the reduced tongue correctness at Level 2 compared with Level 1 during rapid continuous movement (Table 4 the results suggested that the tongue can move more rapidly in the horizontal direction but the accuracy is compromised compared with the protrusion/retraction direction during continuous movement. Further studies are warranted to examine the potential factors that may influence these differences in tongue movement speed (*e.g*., speed of retraction, speed of change in direction) depending on the direction and continuousness of the movement. The greater CV of time interval at Level 1 compared with Levels 2 and 3 (Figure 5) would be due to the absence of a physical stopper (tooth or key) in Level 1. The increased CV of time interval during the concurrent motor task compared with other tasks at Level 3 implied that this task was challenging for both hand and tongue.

The current study focused on comparing the presence of significant main effects of an additional concurrent task on hand and tongue motor performance. Statistical comparison of the absolute values of the dependent variables between the hand and tongue performance was not conducted because the measurement technique and the task details were not regarded comparable enough to allow for the standard statistical comparisons in a strict sense. Nonetheless, every effort was provided to make the measurement technique and task as similar as possible within the constrained technical and practical allowance. Despite these constraints, the current study revealed novel and important uniqueness of dual task performance that involves tongue motor control: the susceptibility to interference from a concurrent task is different between hand and tongue depending on the type of a concurrent task (*i.e*. motor or cognitive). In practice, it would be advisable that the task in which rapid reaction is critical should be allocated to the tongue in a situation where both tongue and hand are concurrently used. In a situation with concurrent allocation of rapid motor tasks between the tongue and hand, the task in which the reaction time is crucial should be allocated to the tongue rather than the hand. In a situation with concurrent cognitive and rapid motor tasks, that kind of task should be allocated to the hand rather than the tongue. This way, the findings would help the potential users to practically decide the allocation of TDS depending on their specific needs and conditions that might involve dual task.

Tongue movement is controlled with coordinated activation of bilateral muscles that are directly innervated from the brain stem with the cranial nerves (hypoglossal and vagus nerves). The finger movement is controlled with unilateral forearm and hand muscles that are innervated with the ulnar, median, and radial nerves through the spinal cord. The shorter length of the nerves from the brain stem to the tongue muscles compared with the hand and forearm muscles may in part contribute to the smaller susceptibility to dual-task interference in the tongue than hand during the reaction task and the rapid motor task. Compared with finger movement, even simple tongue movement requires a greater engagement of larger area and volume of brain structures, including greater activation of the bilateral postcentral gyrus, supplementary motor area, and anterior cingulate cortex [29]. Supplementary motor area and anterior cingulated cortex are involved in the planning, initiation, and execution of movement as well as the allocation of attention [30-33]. The greater effect of a concurrent tongue than hand task on cognitive correctness may be related to the greater engagement of these brain structures during tongue movement.

## Conclusions

In conclusion, the influence of an additional concurrent task on motor performance was distinct between the hand and tongue for rapid movement but not for slow movement. In rapid movement with a concurrent motor task, most aspects of motor performance were degraded in the hand, while tongue speed during rapid continuous task was maintained. With a concurrent cognitive task, most aspects of motor performance were degraded in the tongue, while hand accuracy during the rapid discrete task and hand speed during the rapid continuous task were maintained. The results indicated that rapid hand and tongue movements are more consistently susceptible to interference from concurrent motor and cognitive tasks, respectively, compared with the other task.

## List of abbreviations

ANOVA: Analysis of Variance; CV: Coefficient of Variation; GUI: Graphical User Interface; H: Hand (task); HC: concurrent Hand and Cognitive (task); HT: concurrent Hand and Tongue (task); LED: Light Emitting Diode; PCB: Printed Circuit Board; RMSE: Root Mean Square Error; SSP: Sensor Signal Processing; T: Tongue (task); TC: concurrent Tongue and Cognitive (task); TDS: Tongue Drive System.

## Competing interests

The authors declare that they have no competing interests.

## Authors' contributions

MG and MS designed, directed, and supervised the study. XH and ANJ prepared the setup for Tongue Drive System and hand, respectively. XH and ANJ carried out the experiments with a greater responsibility to XH for Session 1 and ANJ for Session 2. MS played a major role in interpreting the findings. All authors contributed to the development of the manuscript. All authors read and approved the final manuscript.

## Authors' information

ANJ is a Ph.D. candidate in the School of Electrical and Computer Engineering at the Georgia Institute of Technology. She received a B.S. in Electrical Engineering from Florida A&M University and a M.S. in Electrical and Computer Engineering from Georgia Institute of Technology. Currently, she conducts research in the Neuromuscular Physiology Lab and Intelligent Control Systems Laboratory modeling the effects of aging and dual tasks on motor control. She is a student member of the Institute of Electrical and Electronics Engineers (IEEE), the IEEE Engineering in Medicine and Biology Society (EMBS), and the Society for Neuroscience.

XH received his B.S. and M.S. in Mechanical Engineering from Tsinghua University, China. He is currently pursuing his Ph.D. in Electrical and Computer Engineering at Georgia Institute of Technology. Meanwhile, he is working as a Research Assistant in the GTBionics Lab, focusing on research and development of advanced assistive devices. He is a student member of the Institute of Electrical and Electronics Engineers (IEEE) and the Engineering in Medicine and Biology Society (EMBS).

MG holds a Ph.D. in Electrical Engineering and Computer Science from University of Michigan, Ann Arbor, MI. His current academic positions include Assistant Professor at School of Electrical and Computer Engineering, where he holds the ON Semiconductor Junior Faculty Chair position and Director of the GT-Bionics Lab at Georgia Institute of Technology. He is a Senior member of the Institute of Electrical and Electronic Engineers (IEEE) and serves as an Associate Editor for the *IEEE Transactions on Circuits and Systems II (Express Briefs) *and *IEEE Transactions on Biomedical Circuits and Systems*.

MS holds a Ph.D. in Multidisciplinary Sciences (Biomechanics and Exercise Physiology) from University of Tokyo. His current academic positions include Associate Professor at School of Applied Physiology and Director of Neuromuscular Physiology Lab at Georgia Institute of Technology, Research Physiologist at VA Rehabilitation R&D Center of Excellence in Atlanta, and Adjunct Associate Professor at Department of Physiology and Department of Rehabilitation Medicine at Emory School of Medicine. He is a Fellow member of the American College of Sports Medicine and serves as an Associate Editor for *Medicine and Science in Sports and Exercise *and Editorial Board member for *Journal of Applied Physiology *and *Journal of Electromyography and Kinesiology*.
